# Impulse oscillometry system and pulmonary function test assessment of the impact of tumor location, staging, and pathological type on lung function in primary lung cancer

**DOI:** 10.1186/s12890-024-03363-5

**Published:** 2024-11-11

**Authors:** Jia Li, Xiaoxu Wan

**Affiliations:** 1 Department of Radiation Oncology, Shandong Cancer Hospital and Institute, Shandong First Medical University, Shandong Academy of Medical Sciences, Jinan, Shandong Province China; 2 Department of Special Inspection Section, Shandong Cancer Hospital and Institute, Shandong First Medical University, Shandong Academy of Medical Sciences, Jinan, Shandong Province China

**Keywords:** Primary, Lung function, Pulmonary function test (PFT), Impulse oscillometry system (IOS)

## Abstract

**Purpose:**

To study the effects of tumor site, stage, pathologic type and imaging findings on lung function in primary lung cancer, as well as the correlation between impulse oscillometry system (IOS) and pulmonary function test (PFT) parameters.

**Methods:**

The impact of tumor location, staging, and pathological type on lung function were evaluated in 219 patients with primary lung cancer through IOS and PFT. Spearman correlation coefficient was used to analyze the relationship between IOS parameters and PFT parameters.

**Results:**

The PFT parameters in adenocarcinoma were significantly higher than those in SCLC, while the other parameters in IOS were obviously lower than those in SCLC except X_5Hz_ (*P* < 0.05). The PFT parameters of FVC%, FEV1% and MVV% in SCC were evidently higher than those in SCLC, while the parameters of IOS were significantly lower than those in SCLC (*P* < 0.05). The PFT parameters of adenocarcinoma were higher than those of SCC (*P* < 0.05). In the PFT parameters of stage I patients, FEV1/FVC%, MEF50%, MMEF75/25%, and DLCO% were markedly higher than those of stage II patients, and FVC%, FEV1%, FEV1/FVC%, MEF50%, MEF25%, PEF%, MMEF75/25%, TLC%, and DLCO% were obviously higher than those of stage III and IV patients; and the MVV%, Z_5Hz_%, R_5Hz_% in IOS parameters were obviously lower than those in stage III, while Fres (1/s) and X_5Hz_ were significantly lower than those in stage IV (*P* < 0.05). Compared with Phase IV, the X_5Hz_ of stage II patients was clearly higher (*P* = 0.023). Besides, PFT parameters of peripheral lung cancer were obviously higher than those of central lung cancer (*P* < 0.05), while Z_5Hz_%, Fres (1/s) and R_5Hz_% of IOS parameters were clearly lower than those of central lung cancer (*P* < 0.05). Moreover, for patients without and with other pulmonary imaging manifestations, the PFT parameters of the former were significantly higher than those of the latter (*P* < 0.05), while only Fres (1/s) of IOS parameters was significantly lower than the latter (*P* < 0.05). Furthermore, there is a low to moderate correlation between IOS parameters and PFT parameters.

**Conclusion:**

Patients with central SCLC and SCC and advanced lung cancer had the worst lung function. The IOS parameters show a good correlation with the traditional PFT parameters, and IOS can be used as an alternative measurement method for PFT when necessary.

**Supplementary Information:**

The online version contains supplementary material available at 10.1186/s12890-024-03363-5.

## Introduction

Lung cancer is one of the leading causes of cancer deaths worldwide, accounting for 11.4% of all cancer patients and 18.0% of deaths [1]. In China, the incidence of lung cancer has increased rapidly in recent years [2]. At present, surgical resection, chemotherapy, radiotherapy, molecular targeted therapy and immunotherapy are still the main treatment methods [3–5]. However, surgery, radiotherapy and drug therapy can easily cause lung injury [6, 7], which often leads to the aggravation of clinical symptoms. Therefore, it is particularly important to evaluate the pulmonary function of patients before treatment.

According to the recommendations of the American Thoracic Society (ATS) and the European Respiratory Society (ERS), spirometry is the most commonly used method to evaluate lung function, which is helpful for the diagnosis and monitoring of obstructive and restrictive diseases [8]. However, due to the specific respiratory maneuvers required for lung capacity measurement, not every patient can easily follow instructions to obtain satisfactory results, especially children, elderly people, and patients with cognitive impairment.

Impulse oscillometry system (IOS) is a non-invasive and simple method for diagnosing lung diseases. It uses soundwaves to measure airway resistance, and does not rely on patient cooperation, making it particularly suitable for children and the elderly [9]. In recent years, studies have found that IOS is more sensitive than spirometry in assessing small airway obstruction, and the degree of airway obstruction is closely related to spirometry [10, 11]. Some researchers believe that IOS is effective in diagnosing small airway and obstructive diseases in patients. When forced vital capacity cannot be performed or there are contraindications to perform the FVC maneuver, IOS can become a suitable method for measuring lung function [12, 13]. IOS is a variant of forced oscillation measurement technology (FOT), characterized by sending pressure pulse waves to the lungs, and then applying fast Fourier transform to decipher data, so that impedance can be calculated at multiple frequencies [14]. Based on these measurement results, the impedance value (Zrs) of the respiratory system can be calculated, which is the sum of resistance (Rrs) and reactance (Xrs) [14]. The reactance at 5 Hz (X_5Hz_) reflects the peripheral elasticity of the lungs (the capacitance energy of the lungs). Diseases that affect lung elasticity (such as interstitial lung disease) can negatively increase capacitance, making the X_5Hz_ value more negative. X_5Hz_ can also provide important information about small airways [14, 15]. The increase of R_5Hz_ parameter greater than R_20Hz_ parameter indicates small airway disease, and the simultaneous increase of these two parameters indicates proximal airway disease [14, 16]. In addition, studies have found that the value of Fres may be higher in restrictive and obstructive pulmonary disease [14, 16].

Therefore, the aim of this study is to retrospectively analyze the IOS and pulmonary function test (PFT) data of 219 patients with lung cancer to explore the effects of tumor location, stage, pathological type and imaging findings on lung function, and to reveal the relationship between IOS and PFT parameters.

## Methods

### Subjects

Retrospective analysis between August 2020 and February 2021 in Shandong province the first medical university affiliated tumor hospital clinical data of patients with primary lung cancer.

Inclusion criteria: (1) Meet the diagnostic criteria in the “Guidelines for the diagnosis and treatment of primary lung cancer (2022 edition)” [2]; (2) Not receiving any anticancer drugs or drugs affecting blood picture and immune function; (3) No obvious abnormalities of heart, liver, kidney and bone marrow function; (4) The patient has clear awareness and is able to complete pulmonary function tests.

Exclusion criteria: (1) Patients with contraindications to pulmonary ventilation function testing; (2) Pregnant or lactating women.

### PFT

PFT was measured by Jaeger MasterScreen PFT System (Germany), and the data of forced vital capacity (FVC), forced expiratory volume in 1 s (FEV1), FEV1/FVC, peak expiratory flow (PEF), maximal expiratory flow at 25% of FVC (MEF50), maximal expiratory flow at 50% of FVC (MEF25), maximal mid-expiratory flow at 75/25% of FVC (MMEF75/25), maximum voluntary ventilation (MVV), total lung capacity (TLC), diffusing capacity of the lungs for carbon monoxide (DLCO) and other were collected. The quality control standard of PFT measurement was adopted “Standardization of Spirometry: 2019 Update” recommended by ATS/ERS [8]. Because the normal reference values of PFT were different in patients with different age, sex, height, weight, and races, all indicators were analyzed using the percentage of measured values to expected values [17].

### IOS

IOS measurement was performed using the Jaeger MasterScreen IOS System, and data such as the patient’s resonance frequency (Fres), impedance at 5 Hz (Z_5Hz_), Resistance at 5 Hz(R_5Hz_), Resistance at 10 Hz(R_10Hz_), Resistance at 15 Hz(R_15Hz_), Resistance at 20 Hz(R_20Hz_) and Reactance at 5 Hz (X_5Hz_) were collected. At present, there is no uniform quality control standard and normal reference range for IOS in the world. This study adopted the IOS quality control standard formulated by the Working Committee of Lung Function and Clinical Respiratory Physiology, Chinese Society of Pulmonologists, Chinese Medical Doctor Association. Except for Fres and X_5Hz_, which were analyzed using measured values, all other indicators were analyzed using the percentage of measured values to expected values [18].

### Statistical analysis

SPSS 27.0 software (IBM, New York, USA) was used for statistical analysis. The measurement data were expressed as mean ± standard deviation (x ®±s), and the Shapiro-Wilk (S-W) method was used to test for normal distribution. The data conforming to normal distribution were tested by two independent samples t test. Non-compliance with normal distribution was tested using Kruskall-Wallis for multiple component parametric tests and Mann-Whitney for two independent samples nonparametric tests. Count data were expressed as rate (%), and the differences between groups were compared by chi-square test and Fisher’s exact test. Spearman correlation coefficient analysis was used to determine the relationship between IOS and PFT parameters. This study used the following parameters as critical values: 0<| r |<0.3 is weakly correlated; 0.3<| r |<0.7 is moderately correlated| R |>0.7 is strongly correlated [19]. The risk factors for lung function in lung cancer patients were analyzed using multivariate logistic regression analysis. Statistical significance was accepted as *P* < 0.05.

## Results

### Comparison of clinic pathological characteristics of patients

This study included 219 patients with lung cancer, including 133 cases of male, 86 cases of women. The age ranged from 26 to 88 years, with an average age of (61.02 ± 9.95) years and a median age of 62 years. There were 27 cases of small cell lung cancer (SCLC), 37 cases of squamous cell carcinoma (SCC), and 155 cases of adenocarcinoma. According to the eighth edition of the TNM staging criteria for lung cancer released by the International Union Against Cancer (UICC) in 2017 [20], there were 102 cases in stage I, 24 cases in stage II, 49 cases in stage III, and 44 cases in stage IV. According to chest thin-section CT or fiberoptic bronchoscopy, 156 cases were peripheral lung cancer and 63 cases were central lung cancer. Among them, there were 88 cases with pulmonary symptoms such as obstructive pneumonia and atelectasis on imaging, and 131 cases without the above symptoms on imaging. The clinical characteristics of the patients in each group are compared in Table 1. The three groups of patients were significantly correlated with gender, age, smoking history, TNM stage, tumor location and imaging manifestations (*P* < 0.001), but not with height, weight, past medical history (*P* > 0.05).

**Table 1:** Correlation between clinicopathological features in 219 lung cancer patients

Features	Adenocarcinoma (*n* = 155)	SCC (*n* = 37)	SCLC (*n* = 27)	X^2^ value	Fisher value	*P* value
Gender Male Female	75 80	37 0	21 6		45.854	< 0.001
Age (years) < 60 ≥ 60	78 77	5 32	9 18	17.564		< 0.001
Height (cm) < 165 ≥ 165	91 64	17 20	16 11	2.068		0.373
Weight (kg) < 65 ≥ 65	70 85	14 23	13 14	0.835		0.683
Smoking history Yes No	115 40	10 27	10 17	35.991		< 0.001
Past medical history				5.882^a^		0.258
Body health	102	17	18			
hypertension, CHD, diabetes	46	16	7			
Respiratory diseases	7	4	2			
TNM stage I II III IV	93 13 17 32	8 9 17 3	1 2 15 9	68.515^a^		< 0.001
Tumor location Peripheral lung cancer Central type lung cancer	135 20	13 24	8 19	65.370		< 0.001
Imaging manifestations No Other symptoms in the lungs	116 39	5 32	10 17	53.384		< 0.001

Note: CHD: Coronary heart disease; ^a^ corrected X2 statistic. Other symptoms in the lungs: obstructive pneumonia, atelectasis and other radiological findings

### Effects of different pathological types on lung function

In this study, the impact of different pathological types on lung function in 219 lung cancer patients was shown in Table 2. The PFT parameters of adenocarcinoma were significantly higher than those of SCLC, and the IOS parameters except for X5Hz were significantly lower than those of SCLC (*P* < 0.05). In addition, the PFT parameters of SCC except DLCO were higher than those of SCLC, but only FVC%, FEV1%, and MVV% were significantly different (*P* < 0.05), and the IOS parameters of SCC were significantly lower than those of SCLC (*P* < 0.05). Moreover, the PFT parameters of adenocarcinoma were significantly higher than those of SCC (*P* < 0.05), while the difference in IOS parameters between adenocarcinoma and SCC was not statistically significant (*P* > 0.05). Furthermore, the normal distribution test results for different pathological types were shown in Supplementary Table 1.

**Table 2:** Influence of different pathological types on pulmonary function in 219 patients with lung cancer ( x ®±s)

Parameters	Adenocarcinoma( *n* = 155)	SCC ( *n* = 37)	SCLC ( *n* = 27)	t/Z value	*P* value
FVC,%pred.	107.20 ± 1.43	96.32 ± 3.33	85.48 ± 3.49	5.828^a^ 2.204^a^ 3.248^a^	< 0.001^c^ 0.031^d^ 0.001^e^
FEV1,%pred.	99.41 ± 1.59	84.08 ± 4.05	70.87 ± 4.24	-5.512^b^ -1.964^b^ -3.671^b^	< 0.001^c^ 0.049^d^ < 0.001^e^
FEV1/FVC,%	96.58 ± 0.82	88.62 ± 2.16	84.31 ± 2.92	-4.699^b^ -1.210^b^ -3.635^b^	< 0.001^c^ 0.226^d^ < 0.001^e^
MEF50,%pred.	73.72 ± 2.32	50.08 ± 4.49	36.78 ± 3.93	-5.805^b^ -1.781^b^ -4.422^b^	< 0.001^c^ 0.075^d^ < 0.001^e^
MEF25,%pred.	55.58 ± 2.14	41.80 ± 3.53	35.65 ± 4.92	-4.230^b^ -1.665^b^ -3.329^b^	< 0.001^c^ 0.096^d^ 0.001^e^
PEF,%pred.	95.23 ± 1.59	80.41 ± 4.03	69.56 ± 4.02	-5.241^b^ -1.726^b^ -3.033^b^	< 0.001^c^ 0.084^d^ 0.002^e^
MMEF75/25,%pred.	65.69 ± 2.03	46.33 ± 3.99	35.79 ± 3.86	-5.284^b^ -1.767^b^ -4.187^b^	< 0.001^c^ 0.077^d^ < 0.001^e^
MVV,%pred.	97.17 ± 1.84	80.01 ± 3.27	67.76 ± 3.93	6.250^a^ 2.405^a^ 4.193^a^	< 0.001^c^ 0.019^d^ < 0.001^e^
TLC,%pred.	100.25 ± 1.06	94.31 ± 2.16	88.23 ± 2.64	4.329^a^ 1.794^a^ 2.455^a^	< 0.001^c^ 0.078^d^ 0.015^e^
DLCO,%pred.	98.48 ± 1.40	82.82 ± 3.58	82.57 ± 4.14	4.231^a^ 0.046^a^ 4.683^a^	< 0.001^c^ 0.964^d^ < 0.001^e^
Z_5Hz_[kPa/(L/s)],%pred.	95.36 ± 2.40	101.69 ± 7.45	126.37 ± 7.10	-4.333^b^ -2.875^b^ -0.186^b^	< 0.001^c^ 0.004^d^ 0.852^e^
F_res_(1/s)	13.08 ± 0.37	14.45 ± 0.91	17.67 ± 0.95	-4.404^b^ -2.746^b^ -1.246^b^	< 0.001^c^ 0.006^d^ 0.213^e^
R_5Hz_[kPa/(L/s)],%pred.	92.24 ± 2.21	95.73 ± 6.25	120.7 ± 6.68	-4.228^b^ -2.909^b^ 0.000^b^	< 0.001^c^ 0.004^d^ 1.000^e^
R_10Hz_[kPa/(L/s)],%pred.	89.90 ± 1.90	88.81 ± 4.47	109.98 ± 5.04	-4.000^a^ -3.124^a^ 0.243^a^	< 0.001^c^ 0.003^d^ 0.809^e^
R_15Hz_[kPa/(L/s)],%pred.	88.77 ± 1.81	86.59 ± 3.87	103.87 ± 4.57	-3.183^a^ -2.889^a^ 0.523^a^	0.002^c^ 0.005^d^ 0.602^e^
R_20Hz_[kPa/(L/s)],%pred.	92.96 ± 1.98	89.70 ± 3.84	104.33 ± 4.27	-2.241^a^ -2.530^a^ 0.731^a^	0.026^c^ 0.014^d^ 0.466^e^
X_5Hz_[kPa/(L/s)]	-0.09 ± 0.00	-0.45 ± 0.35	-0.12 ± 0.01	-3.108^b^ -2.315^b^ -0.522^b^	0.002^c^ 0.021^d^ 0.602^e^

Note: ^a^ t value; ^b^ Z value; ^C^ Adenocarcinoma vs. SCLC; ^d^ SCC vs. SCLC; ^e^ Adenocarcinoma vs. SCC

### Effects of different TNM stages on lung function

The impact of different TNM stages on lung function in 219 lung cancer patients was shown in Table 3. The PFT parameters of stage I patients were slightly higher than those of stage II, and there were significant differences in FEV1/FVC%, MEF50%, MMEF75/25%, and DLCO% (*P* < 0.05); However, there was no statistically significant difference in IOS parameters between stage I and stage II patients (*P* > 0.05). The PFT parameters of stage I patients were higher than those of stage III and IV patients, and there were significant differences in FVC%, FEV1%, FEV1/FVC%, MEF50%, MEF25%, PEF%, MMEF75/25%, TLC%, and DLCO% (*P* < 0.05). Except for X_5Hz_, the IOS parameters of stage I patients were all lower than those of stage III and IV patients, and MVV%, Z_5Hz_%, and R_5Hz_% showed statistically significant differences between stage I and III (*P* < 0.05). Fres (1/s) and X_5Hz_ between stage I and stage IV patients were obviously different (*P* < 0.05). There was no obvious difference in R_10Hz_%, R_15Hz_% and R_20Hz_% (*P* > 0.05). The PFT parameters of stage II patients were slightly higher than those of stage III patients except for FEV1/FVC%, while the IOS parameters were lower than those of stage III patients except for X_5Hz_, but the differences were not statistically significant (*P* > 0.05). In addition, the PFT parameters of stage II patients were higher than those of stage IV patients, while the IOS parameters except for X_5Hz_ were lower than those of stage IV patients, and only X_5Hz_ showed significant differences (*P* < 0.05). Moreover, there was no statistically significant difference in PFT and IOS parameters between stage III patients and stage IV patients (*P* > 0.05). Furthermore, the results of the normal distribution test for different TNM stages were shown in Supplementary Table 2.

**Table 3:** Influence of different TNM stages on pulmonary function in 219 patients with lung cancer (x ®±s)

Parameters	I stage (*n* = 102)	II stage (*n* = 24)	III stage (*n* = 49)	IV stage (*n* = 44)	t/Z value	*P* value
FVC,%pred.	107.77 ± 1.60	104.34 ± 3.10	96.92 ± 2.87	96.42 ± 3.83	0.944^a^ 3.557^a^ 2.737^a^ 1.596^a^ 1.608^a^ 0.105^a^	0.347^c^ 0.001^d^ 0.008^d^ 0.115^e^ 0.113^f^ 0.917^g^
FEV1,%pred.	100.25 ± 1.87	92.15 ± 4.37	86.99 ± 3.17	84.85 ± 4.43	-1.743^b^ -3.698^b^ -2.802^b^ -1.174^b^ -0.898^b^ -0.339^b^	0.081^c^ < 0.001^d^ 0.005^d^ 0.240^e^ 0.369^f^ 0.735^g^
FEV1/FVC,%	96.82 ± 1.01	90.73 ± 3.04	92.18 ± 1.52	89.87 ± 2.27	-2.392^b^ -2.959^b^ -2.992^b^ -0.041^b^ -0.116^b^ -0.165^b^	0.017^c^ 0.003^d^ 0.003^d^ 0.967^e^ 0.908^f^ 0.869^g^
MEF50,%pred.	75.29 ± 2.81	57.21 ± 6.01	56.01 ± 4.03	56.28 ± 5.07	-2.724^b^ -4.169^b^ -3.649^b^ -0.188^b^ -0.186^b^ -0.042^b^	0.006^c^ < 0.001^d^ < 0.001^d^ 0.851^e^ 0.852^f^ 0.966^g^
MEF25,%pred.	56.15 ± 2.74	52.22 ± 6.20	46.49 ± 3.50	42.39 ± 3.37	-0.960^b^ -2.395^b^ -2.700^b^ -0.734^b^ -1.161^b^ -0.685^b^	0.337^c^ 0.017^d^ 0.007^d^ 0.463^e^ 0.245^f^ 0.493^g^
PEF,%pred.	95.13 ± 2.06	87.88 ± 4.35	84.68 ± 3.29	82.99 ± 3.72	-1.305^b^ -2.744^b^ -2.911^b^ -0.804^b^ -0.886^b^ -0.073^b^	0.192^c^ 0.006^d^ 0.004^d^ 0.421^e^ 0.376^f^ 0.942^g^
MMEF75/25,%pred.	66.97 ± 2.51	53.78 ± 5.49	51.64 ± 3.56	50.22 ± 4.14	2.268^a^ 3.496^a^ 3.573^a^ 0.334^a^ 0.514^a^ 0.262^a^	0.025^c^ 0.001^d^ < 0.001^d^ 0.739^e^ 0.609^f^ 0.794^g^
MVV,%pred.	95.50 ± 2.24	89.68 ± 4.68	84.55 ± 3.34	86.69 ± 4.36	1.132^a^ 2.754^a^ 1.981^a^ 0.885^a^ 0.437^a^ -0.393^a^	0.260^c^ 0.007^d^ 0.050^d^ 0.379^e^ 0.664^f^ 0.695^g^
TLC,%pred.	101.59 ± 1.09	98.05 ± 2.41	94.49 ± 2.10	92.38 ± 2.60	1.393^a^ 2.997^a^ 3.265^a^ 1.032^a^ 1.599^a^ 0.638^a^	0.166^c^ 0.004^d^ 0.002^d^ 0.306^e^ 0.115^f^ 0.525^g^
DLCO,%pred.	100.46 ± 1.51	89.80 ± 4.45	88.76 ± 3.13	86.53 ± 3.32	-2.277^b^ -3.398^b^ -3.732^b^ -0.429^b^ -0.828^b^ -0.219^b^	0.023^c^ 0.001^d^ < 0.001^d^ 0.668^e^ 0.408^f^ 0.826^g^
Z_5Hz_[kPa/(L/s)],%pred.	94.50 ± 2.93	96.97 ± 8.28	108.24 ± 5.20	106.48 ± 6.08	-0.022^b^ -2.287^b^ -1.356^b^ -1.615^b^ -0.924^b^ -0.454^b^	0.983^c^ 0.022^d^ 0.175^d^ 0.106^e^ 0.355^f^ 0.650^g^
F_res_(1/s)	13.07 ± 0.44	13.27 ± 1.23	14.66 ± 0.72	15.22 ± 0.82	-0.398^b^ -1.908^b^ -2.337^b^ -1.515^b^ -1.604^b^ -0.362^b^	0.691^c^ 0.056^d^ 0.019^d^ 0.130^e^ 0.109^f^ 0.718^g^
R_5Hz_[kPa/(L/s)],%pred.	91.77 ± 2.73	92.16 ± 6.83	104.05 ± 4.85	100.62 ± 5.38	-0.099^b^ -2.178^b^ -1.090^b^ -1.621^b^ -0.821^b^ -0.585^b^	0.921^c^ 0.029^d^ 0.276^d^ 0.105^e^ 0.411^f^ 0.559^g^
R_10Hz_[kPa/(L/s)],%pred.	90.07 ± 2.40	87.65 ± 5.18	96.79 ± 3.77	94.46 ± 4.04	0.434^a^ -1.550^a^ -0.974^a^ -1.406^a^ -1.020^a^ 0.422^a^	0.665^c^ 0.123^d^ 0.332^d^ 0.164^e^ 0.311^f^ 0.674^g^
R_15Hz_[kPa/(L/s)],%pred.	88.99 ± 2.27	85.52 ± 4.92	94.18 ± 3.38	91.43 ± 3.56	0.659^a^ -1.290^a^ -0.586^a^ -1.459^a^ -0.978^a^ 0.560^a^	0.511^c^ 0.199^d^ 0.559^d^ 0.149^e^ 0.331^f^ 0.577^g^
R_20Hz_[kPa/(L/s)],%pred.	93.30 ± 2.45	89.53 ± 4.89	97.60 ± 3.52	93.11 ± 3.61	0.676^a^ -1.001^a^ 0.045^a^ -1.326^a^ -0.588^a^ 0.890^a^	0.500^c^ 0.319^d^ 0.964^d^ 0.189^e^ 0.558^f^ 0.376^g^
X_5Hz_[kPa/(L/s)]	-0.09 ± 0.00	-0.09 ± 0.02	-0.36 ± 0.26	-0.12 ± 0.01	-0.346^b^ -1.450^b^ -2.689^b^ -1.259^b^ -2.266^b^ -1.084^b^	0.730^c^ 0.147^d^ 0.007^d^ 0.208^e^ 0.023^f^ 0.278^g^

Note: ^a^ t value,^b^ Z value. ^C^ I stage vs. II stage, ^d^ I stage vs. III stage/ IV stage, ^e^ II stage vs. III stage, ^f^ II stage vs. IV stage, ^g^ III stage vs. IV stage

### Effects of different tumor sites on lung function

The PFT parameters of peripheral lung cancer patients were higher than those of central lung cancer patients (*P* < 0.05). Besides, except for X_5Hz_, the IOS parameters of peripheral lung cancer patients were lower than those of central lung cancer patients, and the differences in Z_5Hz_%, Fres (1/s), R_5Hz_%, and X_5Hz_ were significant (*P* < 0.05), while the differences in R_10Hz_%, R_15Hz_%, and R_20Hz_% were not statistically significant (*P* > 0.05), as shown in Table 4. Furthermore, the normal distribution test results for different tumor sites are shown in Supplementary Table 3.

**Table 4:** Influence of different tumor sites on pulmonary function in 219 patients with lung cancer (x ®±s)

Parameters	Peripheral lung cancer (*n* = 156)	Central lung cancer (*n* = 63)	t/Z value	*P* value
FVC,%pred.	106.29 ± 1.38	93.75 ± 2.85	3.963^a^	< 0.001
FEV1,%pred.	97.89 ± 1.61	81.94 ± 3.32	-4.461^b^	< 0.001
FEV1/FVC,%	95.56 ± 0.88	89.16 ± 1.77	-3.460^b^	0.001
MEF50,%pred.	71.50 ± 2.36	49.51 ± 3.63	-4.983^b^	< 0.001
MEF25,%pred.	54.18 ± 2.15	42.40 ± 3.13	-3.359^b^	0.001
PEF,%pred.	94.85 ± 1.64	76.46 ± 2.87	-5.112^b^	< 0.001
MMEF75/25,%pred.	64.13 ± 2.05	45.36 ± 3.17	-4.807^b^	< 0.001
MVV,%pred.	95.45 ± 1.87	78.73 ± 2.94	4.799^a^	< 0.001
TLC,%pred.	100.04 ± 1.01	92.12 ± 1.92	3.951^a^	< 0.001
DLCO,%pred.	97.35 ± 1.47	85.27 ± 2.67	4.212^a^	< 0.001
Z_5Hz_[kPa/(L/s)],%pred.	96.62 ± 2.74	109.23 ± 4.53	-2.714^b^	0.007
F_res_(1/s)	13.28 ± 0.39	15.37 ± 0.65	-2.947^b^	0.003
R_5Hz_[kPa/(L/s)],%pred.	93.09 ± 2.46	104.38 ± 4.12	-2.590^b^	0.010
R_10Hz_[kPa/(L/s)],%pred.	90.15 ± 2.01	97.23 ± 3.24	-1.876^a^	0.062
R_15Hz_[kPa/(L/s)],%pred.	88.89 ± 1.89	93.66 ± 2.83	-1.370^a^	0.172
R_20Hz_[kPa/(L/s)],%pred.	92.83 ± 2.03	96.24 ± 2.75	-1.350^b^	0.177
X_5Hz_[kPa/(L/s)]	-0.09 ± 0.01	-0.31 ± 0.20	-2.049^b^	0.040

Note: ^a^ t value; ^b^ Z value

### Influence of imaging findings on lung function

As shown in Table 5, the PFT parameters of patients without accompanying other pulmonary imaging manifestations were significantly higher than those with accompanying other pulmonary imaging manifestations (*P* < 0.05). In addition, the IOS parameters of patients without accompanying other pulmonary imaging manifestations were lower than those of patients with accompanying other pulmonary imaging manifestations, except for R_20Hz_% and X_5Hz_, but only Fres (1/s) showed significant differences (*P* < 0.05). As shown in Table 5, the PFT parameters of patients without accompanying other pulmonary imaging manifestations were significantly higher than those with accompanying other pulmonary imaging manifestations (*P* < 0.05).

**Table 5:** Influence of other pulmonary imaging findings on pulmonary function in 219 patients with lung cancer ($\:\stackrel{-}{\varvec{x}}$±s)

Parameters	Other imaging manifestations of the lungs		t/Z value	*P* value
	Absent (*n* = 131)	Existence (*n* = 88)		
FVC,%pred.	106.95 ± 1.62	96.35 ± 2.13	4.029^a^	< 0.001
FEV1,%pred.	99.29 ± 1.81	84.40 ± 2.56	-4.821^b^	< 0.001
FEV1/FVC,%	96.46 ± 0.90	89.65 ± 1.47	-4.427^b^	< 0.001
MEF50,%pred.	74.89 ± 2.58	50.71 ± 2.88	-5.964^b^	< 0.001
MEF25,%pred.	55.60 ± 2.35	43.64 ± 2.67	-3.964^b^	< 0.001
PEF,%pred.	95.26 ± 1.88	81.07 ± 2.33	-4.506^b^	< 0.001
MMEF75/25,%pred.	66.65 ± 2.27	46.94 ± 2.52	-5.591^b^	< 0.001
MVV,%pred.	96.42 ± 2.15	82.05 ± 2.33	4.434^a^	< 0.001
TLC,%pred.	100.09 ± 1.16	94.31 ± 1.50	3.077^a^	0.002
DLCO,%pred.	99.33 ± 1.46	85.76 ± 2.31	4.971^a^	< 0.001
Z_5Hz_[kPa/(L/s)],%pred.	97.83 ± 2.86	103.86 ± 4.08	-0.989^b^	0.323
F_res_(1/s)	13.15 ± 0.41	14.96 ± 0.57	-2.606^b^	0.009
R_5Hz_[kPa/(L/s)],%pred.	94.68 ± 2.65	98.81 ± 3.59	-0.839^b^	0.402
R_10Hz_[kPa/(L/s)],%pred.	91.62 ± 2.15	93.04 ± 2.85	-0.407^a^	0.684
R_15Hz_[kPa/(L/s)],%pred.	90.24 ± 2.03	90.29 ± 2.53	-0.014^a^	0.989
R_20Hz_[kPa/(L/s)],%pred.	94.44 ± 2.21	92.88 ± 2.46	0.465^a^	0.643
X_5Hz_[kPa/(L/s)]	-0.09 ± 0.01	-0.25 ± 0.15	-1.084^b^	0.278

Note: ^a^ t value, ^b^ Z value

### Multivariate regression analysis of factors affecting lung function in patients with lung cancer

To determine the effects of TNM stage, pathological types and tumor location on lung function in patients with lung cancer, we performed multivariate logistic regression analysis while controlling for confounding factors. Multivariate logistic regression analysis showed that TNM stage, pathological types and tumor location were independent risk factors for lung function in patients with lung cancer (*P* < 0.05, Table 6).

**Table 6:** Multivariate logistic regression analysis of risk factors for lung function in lung cancer patients

Factors	HR	95% CI	*P* value
TNM stage			
I	-	-	-
II	2.016	1.189–3.954	0.032
III	1.665	1.154–2.251	0.003
IV	3.128	1.924–8.697	< 0.001
Pathological types			
Adenocarcinoma	-	-	-
SCC	4.365	2.369–6.987	< 0.001
SCLC	4.787	2.765–8.536	< 0.001
Tumor location			
Peripheral lung cancer	-	-	-
Central type lung cancer	1.43	1.089–1.849	0.009

### Correlation between IOS and PFT parameters

As shown in Table 7, there is a low to moderate correlation between IOS and PFT parameters. Among them, Fres (1/s) has the highest correlation with PFT parameters, especially with FVC%, FEV1%, FEV1/FVC%, MEF50%, MEF25%, PEF%, MMEF75/25%, and MVV%, showing a moderate correlation. There is a weak correlation between R_15Hz_%, R_20Hz_%, X_5Hz_ and PFT parameters. Z_5Hz_% and R_5Hz_% were moderately correlated with FEV1%, PEF% and MVV%, and weakly correlated with other parameters. R_10Hz_% is only moderately correlated with MVV%, and weakly correlated with other parameters (Supplementary Table 4).

**Table 7:** Spearman correlation coefficients of IOS and PFT parameters in 219 patients with lung cancer

Parameters	Z_5Hz_[kPa/(L/s)],%pred.	F_res_(1/s)	*R*_5Hz_[kPa/(L/s)],%pred.	*R*_10Hz_[kPa/(L/s)],%pred.	*R*_15Hz_[kPa/(L/s)],%pred.	*R*_20Hz_[kPa/(L/s)],%pred.	X_5Hz_[kPa/(L/s)]
FVC,%pred.	-0.291^**^	-0.359^**^	-0.274^**^	-0.243^**^	-0.198^**^	-0.164^*^	0.186^**^
FEV1,%pred.	-0.326^**^	-0.464^**^	-0.315^**^	-0.280^**^	-0.226^**^	-0.173^*^	0.225^**^
FEV1/FVC,%	-0.266^**^	-0.354^**^	-0.261^**^	-0.237^**^	-0.205^**^	-0.172^*^	0.167^*^
MEF50,%pred.	-0.291^**^	-0.507^**^	-0.289^**^	-0.244^**^	-0.178^**^	-0.116	0.215^**^
MEF25,%pred.	-0.229^**^	-0.467^**^	-0.227^**^	-0.206^**^	-0.143^*^	-0.091	0.200^**^
PEF,%pred.	-0.327^**^	-0.476^**^	-0.332^**^	-0.290^**^	-0.242^**^	-0.174^**^	0.226^**^
MMEF75/25,%pred.	-0.294^**^	-0.543^**^	-0.291^**^	-0.251^**^	-0.181^**^	-0.111	0.237^**^
MVV,%pred.	-0.370^**^	-0.517^**^	-0.381^**^	-0.328^**^	-0.276^**^	-0.219^**^	0.213^**^
TLC,%pred.	-0.205^**^	-0.256^**^	-0.187^**^	-0.181^**^	-0.146^**^	-0.123	0.155^*^
DLCO,%pred.	-0.168^*^	-0.185^**^	-0.150^*^	-0.114	-0.115	-0.124	0.233^**^

Note: ^**^P<0.01; ^*^P<0.05

## Discussion

In recent years, researchers have increasingly recognized that chronic obstructive pulmonary disease (COPD) and interstitial lung disease (ILD) have a significant impact on lung cancer. The presence of COPD and ILD increases the incidence of postoperative complications in lung cancer and is associated with poor clinical prognosis in patients [21, 22]. In addition, there is a difference in long-term survival rates between occasional and past COPD lung cancer patients, suggesting that the timing of COPD diagnosis should be considered in clinical research on lung cancer [23]. In recent years, a large number of studies have confirmed the effectiveness of IOS in diagnosing and monitoring diseases such as COPD and asthma. For example, when the critical value of Fres is ≥ 17.72 Hz, the sensitivity and specificity of COPD diagnosis are 78.9% and 93.1% [24]. In patients with COPD and long-term smoking, IOS parameters have high diagnostic ability for COPD [25]. In addition, IOS has sensitivity and accuracy in the diagnosis of childhood asthma [26, 27]. It is worth noting that the risk of lung cancer incidence and mortality increased with the severity of lung function damage and the decrease of FEV1 [28]. A meta-analysis found that the incidence rate of lung cancer increased with the decrease of FEV1, especially in women with lung cancer [29]. Another study found that the lower the quartile or decile of FEV1 and FEV1/FVC ratio, the higher the incidence of lung cancer. When the FEV1 value was in the lowest quartile (Q4), the incidence of lung cancer increased significantly regardless of FVC [30]. This will demonstrate the importance of lung function assessment in the diagnosis and prognosis of lung cancer, providing convincing reasons for routine lung function monitoring in high-risk populations. Because IOS measurement is easier to implement and has high sensitivity for airway abnormalities [31], it can make up for the shortcomings of PFT. This study for the first time explored the relationship between IOS parameters and lung function in lung cancer patients, and found a certain negative correlation between IOS and PFT parameters.

The objects of this study are patients with primary lung cancer, all of whom have varying degrees of impairment in lung function [2, 32]. Previous studies have shown that the risk of developing lung cancer increases by 20% for every 10% reduction in FEV1 parameters [33]. Moreover, FEV1 parameter is not only closely related to the mortality of patients with SCLC and advanced NSCLC [34, 35], but also an important predictor and risk factor for postoperative complications in patients with early lung cancer [36]. Scholars have also found that the pretreatment FEV1/FVC ratio is an independent factor related to the survival of patients with SCLC (limited phase), and patients with FEV1/FVC < 0.74 are prone to treatment failure and early death [37]. In addition, some studies have indicated that DLCO, which indicates the efficiency of alveolar capillary membrane for gas exchange, that is, the gas exchange function of the lung, can be used as an important predictor of the incidence of complications after pneumonectomy or chest radiotherapy [38, 39], and low DLCO indicated that the patient needs supplemental oxygen or has a higher risk of pulmonary complications. This study found that patients with SCLC had the worst lung function, followed by patients with SCC, and patients with adenocarcinoma had the best lung function. In addition, PFT and IOS showed consistent and significant differences in distinguishing lung function between adenocarcinoma and SCLC. The differences of pulmonary function between the two groups exist in the whole airway of the lung, and SCLC patients exhibit obstructive ventilation dysfunction. In addition, although the DLCO of SCLC patients is within the normal range, it is lower than that of adenocarcinoma patients, indicating a decrease in their ventilation function. When distinguishing lung function between SCC and SCLC, there were significant differences in IOS parameters, while only FVC%, FEV1%, and MVV% showed differences in PFT, indicating that IOS is more sensitive. Although the IOS parameters of SCLC patients are high, the negative value of X_5Hz_ in SCC patients is larger, indicating higher proximal airway resistance in SCLC patients and more significant peripheral airway resistance in SCC patients. When differentiating lung function between adenocarcinoma and SCC, PFT showed significant difference, while IOS showed no significant difference. The difference of lung function between the two groups may mainly focus on the large airway, and the patients with SCC also show obstructive ventilation dysfunction, and their gas exchange function is lower than that of patients with adenocarcinoma.

This study found that patients with early stage lung cancer had better lung function than patients with middle/advanced stage lung cancer. When using PFT to distinguish, the parameters of stage I patients were significantly different from those of other stages, while there was no significant difference in pair-to-stage II, III, and IV patients, indicating that compared with early stage patients, middle/late stage patients had more severe lung function impairment, showing different degrees of small airway/obstructive ventilation dysfunction and reduced ventilation function. When using IOS differentiation, only Z_5Hz_%, Fres (1/s), R_5Hz_%, and X_5Hz_ showed differences between stage I and III/IV, and stage II and IV, respectively, indicating higher peripheral airway resistance in patients in the middle/late stages. This further explains that small airway damage is more severe in mid to late stage patients, and further confirms the reliability of PFT results.

The location of the tumor (central vs. peripheral) is an independent prognostic factor for lung cancer. The prognosis of patients with peripheral lung cancer and central lung cancer is different, with peripheral-type having a better prognosis in adenocarcinoma, SCC, and SCLC [40, 41]. In this study, we found that the lung function of patients with peripheral lung cancer was better than that of patients with central lung cancer, this is consistent with previous research findings. Notably, the peripheral-type includes the upper lobe, middle lobe, and lower lobe. A large body of evidence confirms that lung cancer occurring in the lower lobe has a worse prognosis compared with the upper lobe [42, 43]. A previous study found that the lower lobe is associated with a higher risk of death and a lower proportion of EGFR mutations [44]. Another study found that compared to other lung lobes, patients with upper lobe tumors have longer PFS and OS [45]. Besides, we found that the lung function of patients without other imaging manifestations of the lung was better than that of patients with other imaging manifestations of the lung, and there were significant differences in PFT parameters. Due to the majority of central type lung cancer being SCC or SCLC [2], this coincides with the impact of pathological classification on lung function in patients. A previous study confirmed that lung function decreased in lung cancer patients after concurrent chemoradiotherapy therapy (CCRT), with increases in Z at 5 Hz, R at 5 Hz, R at 20 H, and X at 5 Hz, indicating small airway dysfunction. It is concluded that longitudinal evaluation of lung function through lung function testing can detect CCRT induced injury before clinical symptoms related to CCRT pulmonary toxicity appear [46]. In this study, when using IOS to distinguish the lung function of patients with peripheral lung cancer and central lung cancer, Z_5Hz_%, Fres (1/s), R_5Hz_% and X_5Hz_ are significantly different, indicating that the difference exists in the peripheral airway, which also confirms the characteristics of ventilation dysfunction in the two groups. Patients with other pulmonary imaging findings have poor pulmonary function, mainly because of advanced TNM stage, bronchial obstruction by the tumor itself, smoking history, chronic obstructive pulmonary disease, etc. When IOS was used to distinguish lung function among patients with or without other lung imaging findings, only Fres (1/s) showed a significant difference, which could only indicate the possibility of pulmonary obstruction.

Moreover, the correlation study between IOS and PFT parameters found that Z_5Hz_%, Fres (1/s) and R5Hz% were strongly correlated with different PFT parameters, respectively. In particular, Fres (1/s) had stronger correlation with FVC%, FEV1%, FEV1/FVC%, MEF50%, MEF25%, PEF%, MMEF75/25% and MVV%. FEV1%, FEV1/FVC%, MEF50%, MEF25%, and MMEF75/25% are recognized as important indicators of airway obstruction or small airway obstruction [17], indicating that Fres (1/s) is also an important indicator of airway obstruction, and may become a potential alternative measurement method for evaluating airway obstruction. Besides, both Z_5Hz_% and R_5Hz_% were significantly correlated with FEV1%, indicating that these two parameters are other biomarkers of airway obstruction, which is similar to previous research results [47]. Lee et al. [48] found that R5% and AX% showed the strongest correlation with conventional PFT parameters, as well as the highest correlation with FEF25-75% and sRaw%. AX% and R5% are the optimal IOS parameters for the diagnostic performance of Bronchiolitis Obliterans (BO). Although the IOS parameters observed in central tumors were higher than those in peripheral tumors, IOS did not sufficiently distinguish between atmospheric and small airways. Noord et al. [49] confirmed as early as 1989 that changes in resistance and reactance cannot be fully explained by the observed increase in lung tissue resistance and decrease in lung compliance, and are not specific to restrictive lung disease, and similar changes have also occurred in obstructive pulmonary disease. Therefore, IOS cannot distinguish between obstructive and restrictive pulmonary diseases like PFT.

This study has some limitations cannot be ignored. Firstly, this is a single center retrospective study with a small study population, which may affect the accuracy of the results. Secondly, the impact of other pathological types of primary lung cancer on lung function has not been analyzed. Third, no more comprehensive analysis or stratification of data on comorbidities such as COPD and ILD has been conducted. Fourth, no additional granularity (e.g., distinguishing between upper and lower lobes) was made with respect to tumor location. Finally, because the IOS is a relatively new lung function testing technology, for different populations of reference unified regulations, there is no unified standard. Therefore, we will conduct a large sample, multi-center, prospective study on the impact of primary lung cancer on lung function, and explore the reference value of IOS in cancer patients.

## Conclusion

In conclusion, patients with central SCLC, SCC, and advanced lung cancer had the worst lung function, especially patients with advanced SCLC. In addition, there were low to moderate correlations between IOS parameters and traditional PFT parameters. Therefore, considering the advantages and correlations of these two different detection methods, both IOS and PFT measurements should be performed simultaneously for lung function assessment in lung cancer patients. In the future, the sample size should be expanded to further explore whether IOS can serve as an alternative method to PFT.

## Electronic supplementary material

Below is the link to the electronic supplementary material.

Supplementary Material 1

## Data Availability

The data presented in this study are available on request from the corresponding author.

## Declarations

### Clinical trial number

This study is a retrospective analysis, so it is not applicable.
