# Supplementary material for: Impulse oscillometry system and pulmonary function test assessment of the impact of tumor location, staging, and pathological type on lung function in primary lung cancer
Source: BMC Pulm Med. 2024 Nov 11;24:563. doi: 10.1186/s12890-024-03363-5 (PMC11556193; doi:10.1186/s12890-024-03363-5)
Supplement: Supplementary file 1 — Supplementary Material 1 [file 12890_2024_3363_MOESM1_ESM.docx]

**Supplementary Table 1 Normal distribution test results for different pathological types**

| Parameters | Adenocarcinoma  (n=155) | | SCC  (n=37) | | SCLC  (n=27) | |
| --- | --- | --- | --- | --- | --- | --- |
|  | S-W value | *P* value | S-W value | *P* value | S-W value | *P* value |
| FVC,%pred. | 0.996 | 0.930 | 0.975 | 0.546 | 0.939 | 0.115 |
| FEV1,%pred. | 0.965 | 0.001 | 0.971 | 0.446 | 0.975 | 0.743 |
| FEV1/FVC,% | 0.824 | < 0.001 | 0.937 | 0.036 | 0.928 | 0.062 |
| MEF50,%pred. | 0.988 | 0.231 | 0.937 | 0.037 | 0.918 | 0.035 |
| MEF25,%pred. | 0.892 | < 0.001 | 0.908 | 0.005 | 0.785 | < 0.001 |
| PEF,%pred. | 0.982 | 0.045 | 0.926 | 0.017 | 0.975 | 0.742 |
| MMEF75/25,%pred. | 0.991 | 0.473 | 0.940 | 0.045 | 0.907 | 0.019 |
| MVV,%pred. | 0.994 | 0.818 | 0.964 | 0.277 | 0.983 | 0.926 |
| TLC,%pred. | 0.988 | 0.215 | 0.968 | 0.352 | 0.966 | 0.512 |
| DLCO,%pred. | 0.984 | 0.064 | 0.987 | 0.934 | 0.977 | 0.798 |
| Z_5Hz_[kPa/(L/s)],%pred. | 0.921 | < 0.001 | 0.844 | < 0.001 | 0.967 | 0.522 |
| F_res_(1/s) | 0.925 | < 0.001 | 0.916 | 0.009 | 0.962 | 0.411 |
| R_5Hz_[kPa/(L/s)],%pred. | 0.950 | < 0.001 | 0.894 | 0.002 | 0.959 | 0.342 |
| R_10Hz_[kPa/(L/s)],%pred. | 0.983 | 0.059 | 0.977 | 0.621 | 0.928 | 0.063 |
| R_15Hz_[kPa/(L/s)],%pred. | 0.990 | 0.363 | 0.990 | 0.975 | 0.936 | 0.097 |
| R_20Hz_[kPa/(L/s)],%pred. | 0.985 | 0.102 | 0.987 | 0.938 | 0.932 | 0.079 |
| X_5Hz_[kPa/(L/s)] | 0.753 | < 0.001 | 0.181 | < 0.001 | 0.939 | 0.112 |

**Supplementary Table 2 Results of Normal Distribution Test for Different TNM Stages**

| Parameters | Ⅰstage  (n=102) | | Ⅱstage  (n=24) | | Ⅲstage  (n=49) | | Ⅳstage  (n=44) | |
| --- | --- | --- | --- | --- | --- | --- | --- | --- |
|  | S-W value | *P* value | S-W value | *P* value | S-W value | *P* value | S-W value | *P* value |
| FVC,%pred. | 0.989 | 0.604 | 0.955 | 0.349 | 0.972 | 0.287 | 0.958 | 0.108 |
| FEV1,%pred. | 0.947 | < 0.001 | 0.914 | 0.044 | 0.980 | 0.556 | 0.967 | 0.245 |
| FEV1/FVC,% | 0.818 | < 0.001 | 0.885 | 0.010 | 0.964 | 0.144 | 0.825 | < 0.001 |
| MEF50,%pred. | 0.983 | 0.229 | 0.951 | 0.279 | 0.938 | 0.012 | 0.953 | 0.073 |
| MEF25,%pred. | 0.838 | < 0.001 | 0.897 | 0.019 | 0.923 | 0.003 | 0.941 | 0.026 |
| PEF,%pred. | 0.976 | 0.062 | 0.917 | 0.050 | 0.976 | 0.397 | 0.973 | 0.372 |
| MMEF75/25,%pred. | 0.982 | 0.179 | 0.947 | 0.233 | 0.960 | 0.095 | 0.962 | 0.159 |
| MVV,%pred. | 0.982 | 0.187 | 0.980 | 0.895 | 0.975 | 0.367 | 0.978 | 0.538 |
| TLC,%pred. | 0.988 | 0.512 | 0.973 | 0.744 | 0.957 | 0.072 | 0.964 | 0.179 |
| DLCO,%pred. | 0.973 | 0.037 | 0.976 | 0.802 | 0.975 | 0.388 | 0.983 | 0.745 |
| Z_5Hz_[kPa/(L/s)],%pred. | 0.914 | < 0.001 | 0.847 | 0.002 | 0.960 | 0.094 | 0.914 | 0.003 |
| F_res_(1/s) | 0.910 | < 0.001 | 0.884 | 0.010 | 0.958 | 0.076 | 0.956 | 0.093 |
| R_5Hz_[kPa/(L/s)],%pred. | 0.934 | < 0.001 | 0.930 | 0.096 | 0.969 | 0.215 | 0.934 | 0.015 |
| R_10Hz_[kPa/(L/s)],%pred. | 0.985 | 0.301 | 0.973 | 0.746 | 0.974 | 0.361 | 0.959 | 0.124 |
| R_15Hz_[kPa/(L/s)],%pred. | 0.987 | 0.412 | 0.989 | 0.992 | 0.985 | 0.790 | 0.966 | 0.214 |
| R_20Hz_[kPa/(L/s)],%pred. | 0.988 | 0.466 | 0.982 | 0.936 | 0.967 | 0.185 | 0.957 | 0.100 |
| X_5Hz_[kPa/(L/s)] | 0.924 | < 0.001 | 0.678 | < 0.001 | 0.148 | < 0.001 | 0.659 | < 0.001 |

**Supplementary Table 3 Results of Normal Distribution Test for Different Tumor Sites**

| Parameters | Peripheral lung cancer (n=156) | | Central lung cancer (n=63) | |
| --- | --- | --- | --- | --- |
|  | S-W stage | *P* stage | S-W stage | *P* stage |
| FVC,%pred. | 0.992 | 0.492 | 0.971 | 0.144 |
| FEV1,%pred. | 0.958 | < 0.001 | 0.981 | 0.453 |
| FEV1/FVC,% | 0.833 | < 0.001 | 0.931 | 0.002 |
| MEF50,%pred. | 0.990 | 0.304 | 0.934 | 0.002 |
| MEF25,%pred. | 0.880 | < 0.001 | 0.911 | < 0.001 |
| PEF,%pred. | 0.977 | 0.010 | 0.960 | 0.041 |
| MMEF75/25,%pred. | 0.990 | 0.364 | 0.937 | 0.003 |
| MVV,%pred. | 0.994 | 0.753 | 0.976 | 0.255 |
| TLC,%pred. | 0.995 | 0.872 | 0.984 | 0.583 |
| DLCO,%pred. | 0.990 | 0.315 | 0.994 | 0.991 |
| Z_5Hz_[kPa/(L/s)],%pred. | 0.888 | < 0.001 | 0.963 | 0.053 |
| F_res_(1/s) | 0.901 | < 0.001 | 0.950 | 0.013 |
| R_5Hz_[kPa/(L/s)],%pred. | 0.928 | < 0.001 | 0.971 | 0.139 |
| R_10Hz_[kPa/(L/s)],%pred. | 0.985 | 0.098 | 0.975 | 0.234 |
| R_15Hz_[kPa/(L/s)],%pred. | 0.993 | 0.593 | 0.974 | 0.211 |
| R_20Hz_[kPa/(L/s)],%pred. | 0.991 | 0.396 | 0.964 | 0.059 |
| X_5Hz_[kPa/(L/s)] | 0.730 | < 0.001 | 0.129 | < 0.001 |

**Supplementary Table 4 Results of Normal Distribution Test for Imaging Manifestations**

| Parameters | Other imaging manifestations of the lungs | | | |
| --- | --- | --- | --- | --- |
|  | Absent (n=131) | | Existence (n=88) | |
|  | S-W stage | *P* stage | S-W stage | *P* stage |
| FVC,%pred. | 0.989 | 0.395 | 0.987 | 0.561 |
| FEV1,%pred. | 0.945 | < 0.001 | 0.993 | 0.936 |
| FEV1/FVC,% | 0.834 | < 0.001 | 0.917 | < 0.001 |
| MEF50,%pred. | 0.984 | 0.133 | 0.959 | 0.007 |
| MEF25,%pred. | 0.881 | < 0.001 | 0.894 | < 0.001 |
| PEF,%pred. | 0.978 | 0.032 | 0.968 | 0.028 |
| MMEF75/25,%pred. | 0.988 | 0.311 | 0.957 | 0.005 |
| MVV,%pred. | 0.991 | 0.524 | 0.986 | 0.479 |
| TLC,%pred. | 0.987 | 0.227 | 0.993 | 0.924 |
| DLCO,%pred. | 0.992 | 0.624 | 0.988 | 0.580 |
| Z_5Hz_[kPa/(L/s)],%pred. | 0.916 | < 0.001 | 0.932 | < 0.001 |
| F_res_(1/s) | 0.925 | < 0.001 | 0.957 | 0.005 |
| R_5Hz_[kPa/(L/s)],%pred. | 0.938 | < 0.001 | 0.960 | 0.008 |
| R_10Hz_[kPa/(L/s)],%pred. | 0.983 | 0.105 | 0.982 | 0.245 |
| R_15Hz_[kPa/(L/s)],%pred. | 0.991 | 0.595 | 0.991 | 0.805 |
| R_20Hz_[kPa/(L/s)],%pred. | 0.990 | 0.452 | 0.987 | 0.499 |
| X_5Hz_[kPa/(L/s)] | 0.721 | < 0.001 | 0.110 | < 0.001 |
